# Prospective Study of Optimal Obesity Index Cutoffs for Predicting Development of Multiple Metabolic Risk Factors: The Korean Genome and Epidemiology Study

**DOI:** 10.2188/jea.JE20110164

**Published:** 2012-09-05

**Authors:** Kwang-Pil Ko, Dae-Kyu Oh, Haesook Min, Cheong-Sik Kim, Jae-Kyung Park, Yeonjung Kim, Sung Soo Kim

**Affiliations:** 1 Department of Preventive Medicine, Gachon University of Medicine and Science, Incheon, Korea; 2 Division of Epidemiology and Health Index, Center for Genome Science, Korea Centers for Disease Control & Prevention, Osong, Korea

**Keywords:** obesity, metabolic risk factors, cohort, Korean

## Abstract

**Background:**

In this prospective cohort study, we estimated the risk of developing more than 1 metabolic risk factor, using different obesity indices. In addition, we investigated the relative usefulness of the obesity indices for predicting development of such risk factors and calculated optimal cutoffs for the obesity indices.

**Methods:**

The cohort comprised 10 038 representative residents of a small city and a rural county who were recruited in 2001–2002. Follow-up examinations were conducted every 2 years. Among the 3857 participants without metabolic syndrome at baseline, 1102 new cases occurred during the 6-year follow-up. Receiver operating characteristic (ROC) curves for the obesity indices were plotted to compare the usefulness of the obesity indices.

**Results:**

The numbers of new cases of multiple metabolic risk factors among people in the highest quintiles of body mass index (BMI), waist circumference (WC), waist-hip ratio (WHR), and waist-height ratio at the baseline examination were 2 to 3 times those in the lowest quintiles. The area under the ROC curve for WHR was significantly higher than that for BMI. The optimal BMI cutoff was 24 kg/m^2^ in men and women, and the optimal WC cutoffs were 80 cm and 78 cm in men and women, respectively.

**Conclusions:**

Both overall obesity and central obesity predicted risk of developing multiple metabolic risk factors, and WHR appeared to be a better discriminator than BMI. To prevent development of metabolic diseases among Koreans, it might be useful to lower the cutoff for abdominal obesity, as defined by WC.

## INTRODUCTION

Metabolic syndrome is a cluster of interrelated risk factors of metabolic origin that is closely linked to the development of atherosclerotic cardiovascular diseases.^1^ The most widely recognized metabolic risk factors are high blood pressure, high plasma glucose, and dyslipidemia. Obesity seems to be the predominant underlying risk factor in the development of metabolic syndrome and other cardiovascular risk factors.^2^^,^^3^

Body mass index (BMI) is widely used as a marker of the severity of obesity. The definitions of overweight and obesity recommended by the World Health Organization (WHO), based on data from Western populations, are a BMI of 25 kg/m^2^ or higher and a BMI of 30 kg/m^2^ or higher, respectively.^4^ Because Asians have a higher body fat percentage than whites at the same BMI level,^5^^,^^6^ lower cutoffs for obesity have been recommended for Asians.^7^ Recently, indices of abdominal obesity such as waist circumference (WC), waist-hip ratio (WHR), and waist-height ratio (WHtR) have been reported to be better discriminators of cardiovascular risk factors than an index of overall obesity such as BMI; however, variations in study design and population ethnicity have led to different conclusions regarding which indices are better discriminators of cardiovascular risk factors.^8^^–^^11^

Most studies of obesity indices have used a cross-sectional design, which has an inherent shortcoming: dependent variables can be affected by independent variables. For example, individuals with obesity-related diseases are likely to put more effort into losing weight, which might lead researchers to incorrect conclusions regarding the relationship between obesity and disease.

Multiple metabolic risk factors of endogenous origin can aggregate in a single individual; indeed, this is the definition of metabolic syndrome.^12^ Therefore, we considered incidence of multiple metabolic risk factors as an outcome variable. The aims of this prospective cohort study were to use different obesity indices to estimate incidence of multiple metabolic risk factors, to compare the usefulness of those obesity indices in predicting risk of developing multiple metabolic risk factors, and to identify optimal cutoffs for the obesity indices.

## METHODS

### Study population and data collection

In 2001, the Korea Centers for Disease Control and Prevention (KCDC) established several types of cohorts as part of the Korean Genome and Epidemiology Study, which was conducted to identify environmental, genetic, and genetic–environmental interaction risk factors for developing major chronic diseases such as hypertension, type 2 diabetes, and metabolic syndrome.

For the first project, a community-based cohort was established in a rural community (Ansung) and an urban community (Ansan). Detailed information on the procedure and design of the Ansung and Ansan cohort was previously reported.^13^^,^^14^ Briefly, the cohort is a prospective cohort of 10 038 men and women aged 40 to 69 years who were recruited from 2001 through 2002. Information on participant general characteristics, past medical history, lifestyle, physical activity, diet, reproductive factors, and psychosocial factors was obtained through structured questionnaire interviews. Anthropometric measurements such as blood pressure, height, weight, waist circumference, and body composition were also obtained. Biochemical assessments of fasting serum glucose (FSG), 75-g oral glucose tolerance test, total cholesterol, triglyceride, and high-density lipoprotein (HDL)-cholesterol, among other variables, were also conducted.

Among the 10 038 participants, those with existing metabolic syndrome (≥2 metabolic risk factors except central obesity; *n* = 5503) were excluded from the analysis. Additionally, participants who did not participate in any follow-up examination (*n* = 591) or who had no information on metabolic risk factors (*n* = 87) were also excluded. Thus, the final study population was 3857. The study protocol was approved by the institutional review board of the KCDC.

### Anthropometric measurements

Each participant’s blood pressure and obesity indices were measured by using a standardized protocol. During the initial assessment, systolic and diastolic blood pressures were measured from both arms with the participant in a sitting position, and systolic and diastolic blood pressures were measured twice more from the arm that had the higher systolic blood pressure. Before each measurement, participants rested for at least 5 minutes. The means of the 3 readings from the arm with the higher initial measurement were recorded as the final systolic and diastolic blood pressures. Height and weight were measured using a standardized digital scale.

BMI was calculated as weight in kilograms divided by the square of the height in meters (kg/m^2^). WC in centimeters was measured 3 times at the midpoint between the bottom of the ribs and the top of the iliac crest. The mean of the 3 readings was considered the final WC. Hip circumference in centimeters was measured 3 times at the largest posterior extension of the buttocks, and the mean of the 3 readings was considered the final hip circumference. WHR was calculated as WC divided by hip circumference, and WhtR was calculated as WC divided by height.

### Follow-up and identification of development of multiple metabolic risk factors

Follow-up examinations were conducted every 2 years. The follow-up rates were 86.4%, 75.6%, and 68.8% at the first, second, and third follow-up surveys, respectively. At every follow-up examination, participants were interviewed by using a questionnaire, and blood pressure, FSG, and lipid profile were measured in the same way as at the baseline survey. A participant was considered to have incident multiple metabolic risk factors if at least 2 of the following criteria based on the National Cholesterol Education Program’s Adult Treatment Panel III (NCEP-ATP III)^1^ were met: high blood pressure (systolic blood pressure ≥130 mm Hg, diastolic blood pressure ≥85 mm Hg, or self-reported treatment history for hypertension), hyperglycemia (FSG ≥110 mg/dl or self-reported treatment with antihyperglycemic medication), hypertriglyceridemia (triglyceride ≥150 mg/dl), or low HDL cholesterol (<40 mg/dl for men or <50 mg/dl for women). This definition was also used in similar previous studies to estimate appropriate cutoffs of obesity indices among individuals with multiple metabolic risk factors.^15^^–^^17^

### Statistical analysis

The *t* test or chi-square test was used to test differences between male and female cases and non-cases in the means or proportions of baseline characteristics such as age, obesity indices, blood pressure, FSG, lipid profile, and alcohol intake.

BMI, WC, WHR, and WHtR were divided into quintiles. To determine whether these obesity indices were associated with development of multiple metabolic risk factors, the Cox proportional hazards model was used and modeled for men and women after adjustment for age, residential area, education level, history of cigarette smoking, history of alcohol drinking, and number of metabolic risk factors at baseline. *P* for trend was calculated using the likelihood ratio test.

To identify the obesity index that best predicted development of multiple metabolic risk factors, receiver operating characteristic (ROC) curves were plotted for each obesity index, and the areas under the ROC curve (AUC) were compared among participants who completed the 6-year follow-up (*n* = 3153). False discovery rates (FDR) were calculated to adjust for the increase in α error due to multiple comparisons.

To determine the optimal cutoffs for the obesity indices, the Youden index (sensitivity + specificity − 1) was calculated, and the corresponding value for the maximum of the Youden index was considered the optimal cutoff point. All statistical analyses were conducted using SAS 9.1 (SAS Institute, Cary, NC, USA).

## RESULTS

Table 1 shows the baseline characteristics of the study population. Among male cases, the means for the obesity indices, blood pressure, FSG, and triglyceride were higher, and mean HDL cholesterol level was lower, as compared with male non-cases. Among women, cases also had higher means than non-cases for metabolic risk factors and a lower mean for HDL cholesterol. Among men and women, development of multiple metabolic risk factors was positively associated with residence in a rural area and low education level.

**Table 1.** Baseline characteristics of study population

Variables	Men		*P* value	Women		*P* value
	
	Cases (*n* = 580)	Non-cases (*n* = 1345)		Cases (*n* = 522)	Non-cases (*n* = 1410)	
	Mean ± SD			Mean ± SD		
		
Age	52.8 ± 9.1	51.4 ± 8.9	<0.01	53.1 ± 8.5	48.3 ± 7.7	<0.01
BMI (kg/m^2^)	24.0 ± 2.8	22.8 ± 2.7	<0.01	24.5 ± 3.3	23.7 ± 2.9	<0.01
WC (cm)	83.2 ± 7.2	79.6 ± 7.0	<0.01	81.4 ± 9.1	76.8 ± 8.4	<0.01
WHR	0.90 ± 0.06	0.87 ± 0.06	<0.01	0.88 ± 0.08	0.83 ± 0.08	<0.01
WHtR	0.50 ± 0.04	0.48 ± 0.04	<0.01	0.53 ± 0.06	0.50 ± 0.06	<0.01

	*n* (%)			*n* (%)		
		
Residential area			<0.01			<0.01
Ansung (rural)	335 (35.9)	599 (64.1)		338 (38.9)	530 (61.1)	
Ansan (urban)	245 (24.7)	746 (75.3)		184 (17.3)	880 (82.7)	
Education (years)			0.01			<0.01
0	135 (34.5)	256 (65.5)		251 (40.7)	366 (59.3)	
1–9	335 (30.7)	756 (69.3)		238 (21.1)	891 (78.9)	
≥10	104 (24.5)	320 (75.5)		28 (16.4)	143 (83.6)	
Cigarette smoking			0.21			0.93
Never	106 (26.6)	292 (73.4)		486 (26.7)	1332 (73.3)	
Ex-smoker	178 (30.5)	406 (69.5)		7 (26.9)	19 (73.1)	
Current smoker	293 (31.4)	639 (68.6)		16 (29.1)	39 (70.9)	
Alcohol drinking			0.49			0.32
Never	113 (30.4)	259 (69.6)		354 (28.0)	911 (72.0)	
Ex-drinker	64 (33.9)	125 (66.1)		13 (28.9)	32 (71.1)	
Current drinker	401 (29.6)	953 (70.4)		149 (24.8)	453 (75.3)	
Number of metabolic risk factors			<0.01			<0.01
0	40 (7.33)	506 (92.7)		50 (9.3)	486 (90.7)	
1	540 (39.2)	839 (60.8)		472 (33.8)	924 (66.2)	

BMI: body mass index, WC: waist circumference, WHR: waist-hip ratio, WHtR: waist-height ratio.

*P* values were calculated using the *t* test for continuous variables and the chi-square test for categorical variables.

Table 2 shows the association between levels of obesity indices and incidence of multiple metabolic risk factors. As compared with the lowest quintile, the risk of developing multiple metabolic risk factors linearly increased for ascending quintiles of the obesity indices in both men and women. During the 6-year follow-up, people in the highest quintiles of BMI, WC, WHR, and WHtR at the baseline examination had 2 to 3 times the number of new cases of multiple metabolic risk factors than did the respective lowest quintiles.

**Table 2.** Obesity indices and incidence of multiple metabolic risk factors

	Men					Women				
		
	Range	Cases	No. of subjects	RR^a^ (95% CI)	*P* trend	Range	Cases	No. of subjects	RR^a^ (95% CI)	*P* trend
BMI, kg/m^2^					<0.01					<0.01
Q1	( –20.7)	71	385	Ref.		( –21.4)	85	386	Ref.	
Q2	(20.8–22.3)	103	385	1.61 (1.19–2.18)		(21.5–22.9)	81	387	0.94 (0.69–1.28)	
Q3	(22.4–23.7)	105	385	1.75 (1.29–2.37)		(23.0–24.3)	103	386	1.33 (0.99–1.78)	
Q4	(23.8–25.4)	131	385	2.11 (1.57–2.84)		(24.4–26.1)	112	387	1.31 (0.98–1.75)	
Q5	(25.5– )	170	385	2.79 (2.09–3.72)		(26.2– )	141	386	1.63 (1.24–2.15)	
WC, cm					<0.01					<0.01
Q1	( –74.5)	62	384	Ref.		( –70.3)	53	388	Ref.	
Q2	(74.6–78.6)	88	394	1.44 (1.04–2.00)		(70.4–74.9)	67	371	1.29 (0.90–1.86)	
Q3	(78.7–82.0)	109	375	1.75 (1.28–2.40)		(75.0–79.1)	110	394	1.71 (1.23–2.39)	
Q4	(82.1–86.9)	142	379	2.49 (1.84–3.38)		(79.2–85.0)	128	399	1.59 (1.15–2.21)	
Q5	(87.0– )	179	393	2.88 (2.15–3.86)		(85.1– )	164	380	2.11 (1.53–2.91)	
WHR					<0.01					<0.01
Q1	( –0.825)	50	385	Ref.		( –0.764)	43	386	Ref.	
Q2	(0.826–0.859)	104	385	1.94 (1.38–2.72)		(0.765–0.807)	69	387	1.39 (0.95–2.05)	
Q3	(0.860–0.889)	102	385	1.88 (1.33–2.66)		(0.808–0.856)	98	386	1.58 (1.08–2.32)	
Q4	(0.890–0.926)	143	385	2.66 (1.90–3.73)		(0.857–0.920)	146	387	2.09 (1.42–3.09)	
Q5	(0.927– )	181	385	3.28 (2.33–4.61)		(0.921– )	166	386	2.36 (1.59–3.51)	
WHtR					<0.01					<0.01
Q1	( –0.443)	56	383	Ref.		( –0.452)	46	386	Ref.	
Q2	(0.444–0.471)	87	387	1.52 (1.08–2.13)		(0.453–0.482)	74	387	1.31 (0.91–1.91)	
Q3	(0.472–0.493)	111	386	1.90 (1.38–2.63)		(0.483–0.513)	100	387	1.61 (1.13–2.29)	
Q4	(0.494–0.521)	154	384	2.85 (2.10–3.89)		(0.514–0.555)	133	386	1.86 (1.31–2.64)	
Q5	(0.522– )	172	385	2.79 (2.06–3.79)		(0.556– )	169	386	2.11 (1.49–3.00)	

RR: relative risk, BMI: body mass index, WC: waist circumference, WHR: waist-hip ratio, WHtR: waist-height ratio.

^a^Adjusted for age, residential area, education level, smoking history, alcohol drinking history, and number of metabolic risk factors at baseline.

To identify the obesity index that best predicted development of multiple metabolic risk factors, the ROC curves and the AUC of the obesity indices in relation to multiple metabolic risk factors were plotted and calculated (Figure and Table 3). In predicting multiple metabolic risk factors, the measures of central obesity tended to yield higher AUCs than did BMI. Among men, the AUC for BMI was 0.605 and the AUCs for WC, WHR, and WHtR were 0.646, 0.660, and 0.651, respectively. The AUC for WHR was significantly higher than that for BMI, and the AUCs for WC and WHtR were marginally higher than that for BMI. However, differences among the AUCs for indices of central obesity were not significant. Among women, the AUC for BMI was 0.581, and the AUCs for WC, WHR, and WHtR were 0.657, 0.690, and 0.673, respectively. The AUCs for the indices of central obesity were significantly higher than that for BMI. The differences among the AUCs for indices of central obesity were not significant.

**Figure.**
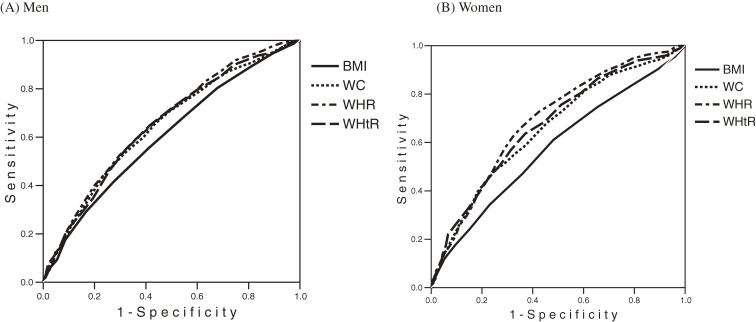
Receiver operating characteristic curves for obesity indices in relation to multiple metabolic risk factors. BMI indicates body mass index; WC, waist circumference; WHR, waist-hip ratio; and WHtR, waist-height ratio.

**Table 3.** AUC of obesity indices in relation to multiple metabolic risk factors

	Men			Women		
		
	AUC (95% CI)	*P* value	FDR	AUC (95% CI)	*P* value	FDR
BMI	0.605 (0.577–0.634)			0.581 (0.551–0.612)		
WC	0.646 (0.618–0.674)	0.046	0.091	0.657 (0.628–0.685)	<0.001	0.001
WHR	0.660 (0.633–0.687)	0.007	0.044	0.690 (0.663–0.717)	<0.001	<0.001
WHtR	0.651 (0.624–0.679)	0.025	0.075	0.673 (0.645–0.701)	<0.001	<0.001

AUC: area under the receiver operating characteristic curve, FDR (false discovery rate): *P* value after adjustment for multiple comparison, BMI: body mass index, WC: waist circumference, WHR: waist-hip ratio, WHtR: waist-height ratio.

*P* values were calculated by testing for difference between the AUC of BMI and those of the indices of central obesity (WC, WHR, and WHtR).

Table 4 shows the optimal cutoffs for predicting incidence of multiple metabolic risk factors. Among men, the Youden index indicated that the optimal BMI cutoff was 24 kg/m^2^ (sensitivity, 0.495; specificity, 0.661) and the optimal WC cutoff was 80 cm (sensitivity, 0.700; specificity, 0.519). Among women, the optimal BMI cutoff was 24 kg/m^2^ (sensitivity, 0.535; specificity, 0.593) and the optimal WC cutoff was 78 cm (sensitivity, 0.623; specificity, 0.6019). Among the measured obesity indices, the Youden index for WHR was highest in men and women.

**Table 4.** Optimal obesity index cutoffs for predicting incidence of multiple metabolic risk factors

	Cutoff	Sensitivity	Specificity	Youden index
Men				
BMI	23 kg/m^2^	0.633	0.519	0.152
	24 kg/m^2^	0.495	0.661	0.156
	25 kg/m^2^	0.341	0.778	0.119
WC	80 cm	0.700	0.519	0.219
	90 cm	0.167	0.918	0.085
WHR	0.89	0.559	0.678	0.237
WHtR	0.49	0.595	0.630	0.225

Women				
	23 kg/m^2^	0.682	0.440	0.122
BMI	24 kg/m^2^	0.535	0.593	0.128
	25 kg/m^2^	0.400	0.715	0.115
WC	78 cm	0.623	0.601	0.224
	80 cm	0.536	0.680	0.216
WHR	0.85	0.648	0.665	0.313
WHtR	0.51	0.613	0.654	0.267

BMI: body mass index, WC: waist circumference, WHR: waist-hip ratio, WHtR: waist-height ratio.

## DISCUSSION

In a community-based cohort of middle-aged Koreans, we found that the presence of overall obesity and central obesity increased the risk of developing multiple metabolic risk factors and that WHR appeared to be a better discriminator than BMI in predicting short-term incidence of multiple metabolic risk factors. The optimal BMI cutoff was 24 kg/m^2^ in both men and women. The optimal WC cutoffs were 80 cm in men and 78 cm in women, which are lower than those specified by current obesity criteria in Korea.^1^

Recent studies have shown that, as compared with Western populations, Asians have less lean muscle mass and more visceral fat mass at a lower BMI and WC.^5^^,^^6^^,^^18^ Therefore, at a given level of BMI, WC, and WHR, the absolute risk of developing metabolic risk factors appears to be higher among Asians than among whites.^19^ Although the WHO has recommended that overweight and obesity in Asian populations be defined as a BMI of 23 kg/m^2^ or higher and a BMI of 25 kg/m^2^ or higher, respectively,^7^ a BMI less than 25 kg/m^2^ was associated with development of multiple metabolic risk factors in our study, especially among men. The International Diabetes Federation (IDF) and the National Cholesterol Education Program (NCEP) suggested that the optimal WC cutoff is 90 cm for men and 80 cm for women in South Asian, Chinese, and Japanese populations.^1^^,^^12^ However, our results showed that a WC between 75 cm and 80 cm increased the risk of developing multiple metabolic risk factors, even though this range is within the defined normal range for WC. When the IDF and NCEP criteria for WC were applied to the present male participants, the sensitivity and specificity in predicting development of multiple metabolic risk factors were 16.7% and 91.8%, respectively. These discrepancies indicate that the WHO, IDF, and NCEP criteria for obesity may not be appropriate for predicting short-term risk of developing metabolic risk factors in middle-aged or elderly Koreans.

Japanese cross-sectional studies of obesity index cutoffs for predicting incidence of multiple metabolic risk factors suggested cutoffs of 24.1 to 24.2 kg/m^2^ for BMI and 85 to 90 cm for WC for men and 23 to 24.7 kg/m^2^ and 78 to 84 cm, respectively, for women.^15^^,^^17^^,^^20^ A Chinese cross-sectional study of obesity index cutoffs for predicting prevalence of multiple metabolic risk factors suggested cutoffs of 24.0 to 24.2 kg/m^2^ for BMI and 80 to 90 cm for WC for men and 24 to 24.7 kg/m^2^ and 80 to 85 cm, respectively, for women.^21^^,^^22^ Previous Korean studies suggested that the WC cutoff for predicting prevalence of multiple metabolic risk factors was 80 to 86 cm in men and 76 to 80 cm in women.^11^^,^^23^^,^^24^ Our results are consistent with those from a previous Korean cross-sectional study based on Korean National Health and Nutrition Examination data in which the study participants were a representative sample population. However, our WC cutoff is somewhat lower than those reported in Japanese and Chinese studies, although the BMI cutoff was similar.

Although many studies have attempted to identify the obesity index that best predicts metabolic risk factors, most such studies were cross-sectional. One meta-analysis suggested that measures of abdominal obesity, and in particular WHtR, are better predictors than BMI of cardiovascular disease risk factors such as hypertension, diabetes, and dyslipidemia^25^; however, except for 1 prospective study, all the evaluated studies were cross-sectional. Huxley et al^19^ systematically reviewed ethnic differences in the cross-sectional relationship among obesity and diabetes and hypertension and found that measures of central obesity were better discriminators of prevalent diabetes and hypertension in Asians and whites.

Although cross-sectional studies of representative populations may have sufficient external validity for the results to be applicable to the general population, the findings of such studies might be biased due to uncertain causal relationships. Previous prospective cohort studies reported somewhat inconsistent results across ethnic groups, and few studies have examined the risk of developing multiple metabolic risk factors. The Health Professionals Follow-Up Study found that WC and BMI were better than WHR in predicting the risk of developing type 2 diabetes in white men.^9^ In Jamaica, WC and BMI were similarly accurate predictors of incident diabetes.^26^ The San Antonio Heart Study suggested that BMI and WC had similar power in predicting development of metabolic syndrome in non-Hispanic whites and Mexican Americans.^27^ In Iranian men and women, WHtR appeared to be better than BMI in predicting the risk of developing type 2 diabetes.^28^^,^^29^ These ethnic differences in results suggest that the predictive power of each obesity index varies by ethnic group.^30^^,^^31^ In our study, waist-related indices were better predictors of metabolic syndrome.

Our study has several limitations. First, our study population might not be a representative sample of the Korean general population, which could restrict the applicability of cutoffs from our study. Second, although obesity indices might change due to lifestyle modification during the follow-up period, only baseline measurements were used in this analysis, and chronological changes in individual obesity were not considered. However, in men, the correlations of BMI at baseline with BMI at 2, 4, and 6 years were 0.94, 0.92, and 0.91, respectively, and the correlations of WC at baseline with WC at each follow-up survey were 0.85, 0.82, and 0.77, respectively (data not shown). In women, the correlations between obesity indices at baseline and obesity indices at each follow-up survey were also high: 0.94, 0.91, and 0.89, respectively, for BMI and 0.81, 0.76, and 0.71, respectively, for WC. Therefore, it is likely that misclassification bias due to change in obesity indices during follow-up had little effect on our study results. Third, although loss to follow-up is inevitable in most cohort studies, it can lead to selection bias, which occurs when loss to follow-up is nonrandom and is related to both the exposure and the outcome.^32^ In our study population, loss to follow-up was positively associated with high obesity index (among men but not women), low education level, and smoking. Unfortunately, we cannot identify whether loss to follow-up was associated with incidence of multiple metabolic risk factors. If it was, relative risk would be affected in women. Nevertheless, our follow-up rate was relatively high; therefore, the effect of selective follow-up is probably limited.

In conclusion, overall obesity and central obesity predicted the risk of developing multiple metabolic risk factors among Koreans, and WHR appeared to be a better discriminator than BMI for predicting the incidence of risk factors. To identify middle-aged and elderly Koreans at high risk of developing multiple metabolic factors, we should consider lowering the current WC cutoffs, especially for men.
